# Pyrolysis of *Aesculus chinensis* Bunge Leaves as for Extracted Bio-Oil Material

**DOI:** 10.3390/polym14225003

**Published:** 2022-11-18

**Authors:** Yiyang Li, Qian Ma, Guanyan Li, Junwei Lou, Xiangmeng Chen, Yifeng He, WanXi Peng

**Affiliations:** 1 Henan Province Engineering Research Center for Forest Biomass Value-Added Products, Henan Agricultural University, Zhengzhou 450002, China; 2 School of Architectural Engineering, Zhejiang Business Technology Institute, Ningbo 315012, China

**Keywords:** *Aesculus chinensis* Bunge leaves, pyrolysis, extract, bio-oil

## Abstract

Biomass rapid pyrolysis technology is easy to implement in continuous production and industrial application, and has become one of the leading technologies in the field of world renewable energy development. Agricultural and forestry waste is an important resource of renewable energy in China. In general, abandoned leaves in forest areas cause serious waste of resources. Its utilization may help to settle the problems of energy deficiency and environment pollution. In this study, *Aesculus chinensis* Bunge leaves (A. Bunge) are used as the research object to study the pyrolysis and extract. The results showed that there are a lot of bioactive components in A. Bunge leaves extract, including acetamide, 5-hydroxymethylfurfural, R-limonene, d-mannose, and dihydroxyacetone. The active components of A. Bunge leaves supply scientific evidence for the exploration and exploitation of this plant. The pyrolysis products of A. Bunge leaves are rich in organic acids, aldehydes, and ketones, which means that A. Bunge leaves can be used as a crude material for the manufacturing of bio-oil or bio-fuel. The pyrolysis products include batilol, pregnenolone, benzoic acid, butyrolactone, and propanoic acid, which can be used in biological medicine, chemical crude materials, and industrial raw material reagents. Therefore, A. Bunge leaves can be used as a good crude material for bio-oil or biofuel production. Combining A. Bunge leaves and fast pyrolysis methods can effectively solve the problem of forestry and agricultural residues in the future.

## 1. Introduction

Every year, a large number of biological wastes are produced in the world, mainly biological solid wastes [1], such as municipal biomass waste, crop waste, livestock feces, and so on, which represent the resource of bioenergy production [2]. At present, the content of biological waste produced in the world is high, and there are some disadvantages in the treatment of biological waste by traditional treatment methods. Direct landfill will produce a large number of pollutants, resulting in soil and groundwater pollution, and its anoxic environment will also lead to the production of a large number of greenhouse gases. Landfill has a low resource utilization rate of biomass waste [3]. Second, incineration will produce a large number of greenhouse gases, resulting in air pollution. Biomass is the largest renewable energy in the world, account for over 10% of energy resources of the whole world [4]. Biomass can be changed into energy products such as bio-oils by thermochemical processes or biolog. In addition to being used as bio-oil, these energy products can also be used as alternative crude material for special lignocellulosic biomass materials such as energy crops [5]. Biodiesel can be prepared by transesterification of soybean oil extracts. Because a large amount of cellulose, hemicellulose, and lignin exist in soybean straw, it can be used to produce various biomaterials, bio-oil, and biochemicals [6,7]. The extraction of bio-oil from wheat straw by low temperature microwave technology provides a new way to save resources [8]. Global biomass production is increasing because bio-oil and biomaterials can replace many commodities produced from fossil resources. The growth of bio-oil and the expansion of the future have been supported by many countries [9].

As a solid form waste material, biological waste is an environmental pollutant that must be disposed of properly. If it is disposed of improperly, it will lead to serious environmental pollution [10]. Because of the huge number of fallen leaves and rotten leaves of trees, garbage dumps cannot be fully received and cannot be burned in large quantities, so landfills must be carried out. Green waste disposal has become a difficulty in production work [11]. In recent years, there are more and more studies on the use of plant leaves to produce high value-added products. The exploitation and utilization of plant leaves can help to protect the environment [12]. Romeo-Guiza et al. found that the crushed leaves can be re-filled into the urban green soil as organic chemical fertilizer. This method not only produces zero pollution gases, but also improves the water storage capacity of the soil [13], effectively increase the air permeability and fertility of soil, truly implement green waste to “turn waste into treasure”, and realize the reuse of resources [14,15]. *Davidia involucrate* leaves are used as raw material to develop a simple, economic, environmental protection biomass rapid production of carbon materials with good electrochemical performance [16]. High performance activated carbon can be prepared from leaves while resources are recovered [17]. Waste sugar cane leaves can be used to produce bio-hydrogen [18]. Moreover, the chemically active ingredients in the leaves are also useful in medicine. For example, chemicals in the leaves of *Dracaena draco* L. have shown to improve insulin resistance in liver cancer cells [19]. *Pentapetes phoenicea* L. leaves can be boiled in water and used to treat adenitis and cold and cough [20].

*Aesculus chinensis* Bunge (A. Bunge) is a tall deciduous tree and is recognized as one of the four beautiful street trees in the world. A. Bunge species are widely distributed around the world with more than 30 species, of which 16 species exist in China [21]. A. Bunge is an important medicinal tree species in China and can be used to treat blood circulation and edema [22]. More than 210 chemical components were isolated from A. Bunge, including other compounds such as triterpenoids, flavonoids, and sterols [23]. Escin has significant antiviral, anti-inflammatory, and other effects, which can be used to study the antitumor activity of bacterial biotransformation products in the human intestine [24]. In recent years, the advantages of A. Bunge resources have been discovered in Xixia County (China). So far, XiXia county has developed A. Bunge nursery base of 4000 mu, stored seedlings of 62 million, annual sales of more than 3 million, and the total benefit of 60 million yuan. It plays a very important role in expanding the forestry industry chain and improving the comprehensive benefits of forestry. However, there is less research on A. Bunge as a potential biomass energy source. The wide range of uses of biomass as a feedstock has been vigorously developed worldwide, and these materials can be converted into different biomass energy sources by biochemical or thermochemical means (combustion, gasification, pyrolysis, and liquefaction) [25]. Pyrolysis as a high-efficiency and high net calorific value method for producing bioenergy, especially in pyrolysis bio-oil, and has attracted great attention from the research society [26].

In this study, A. Bunge leaves were used as the research object, and the pyrolysis and extracts of A. Bunge leaves were studied. In order to explore and optimize the pyrolysis process, this study used PY/GC–MS to identify the pyrolysis components of A. Bunge leaves pyrolysis products and changed the conditions for rapid pyrolysis. The active components of A. Bunge leaves extract from GC–MS were identified. In addition, the functional groups of A. Bunge leaves extract was characterized by FT–IR. This study aims to analyze the development potential of bio-oil. As far as we know, this is also the first time to combine pyrolysis technology with A. Bunge leaves using innovative methods. Developing comprehensive high value-added applications of A. Bunge leaves can promote sustainable utilization and alleviate environmental pollution. The research and development of A. Bunge leaves will supply a substantial scientific basis and rationale in the days to come.

## 2. Material and Methods

### 2.1. Sample Sources

*Aesculus chinensis* Bunge leaves (A. Bunge leaves) were obtained from the Xixia County, Nanyang City in Henan Province. The fresh leaves are first vacuum-dried, then the dried leaves are powdered with a powder beater and sieved with a 120 mesh screen. Active compounds were extracted by mixing approximately 10 g of powder sample with two different organic solvents (ethanol and methanol), the ratio of powder to organic solvent is 1:30.

### 2.2. Experiment Methods

FT–IR Analysis

FT–IR spectra of ethanol and methanol extract samples were detected on Thermo Fisher Scientific iS10 (Waltham, Massachusetts, USA) instrument. A thin potassium bromide (KBr) disk was prepared from a mixture of KBr and the catalyst–wood samples at a ratio of 70:1 using a mortar and pestle. The KBr disk was then loaded into an FTIR spectrophotometer at wavelengths from 400 cm^−1^ to 4000 cm^−1^ for 64 scans [27].

GC–MS Analysis

The chemical composition of ethanol and methanol extract samples from A. Bunge leaves samples were analyzed by GC–MS Agilent 7890B–5977A (California, USA instrument. A HP-5MS column (30 m × 250 μm × 0.25 μm) and an elastic quartz capillary column were used, with a carrier gas of high purity helium at a flow rate of 1 mL/min and a split ratio of 2:1. The temperature program for GC started at 50 °C and increased to 250 °C at a rate of 8 °C/min, followed by a further increase to 280 °C at a rate of 5 °C/min. The entire MS program scanned for a mass range of 30–600 amu with an ionization voltage of 70 eV and an ionization current of 150 μA. The ion source and quadrupole temperatures were set to 230 and 150 °C, respectively [28].

PY/GC–MS Analysis

A. Bunge leaves sample powder was analyzed on pyrolysis CDS 5000–Agilent 7890B–5977A ISQ (California, USA) instrument. The sample was pyrolyzed at 950 °C with a heating rate of 20 °C/MS. The gas produced in the pyrolysis process was then injected into the GC–MS analyzer. The analysis settings for the GC–MS are as follows: TR–5MS column with a capillary size of 0.25 µm × 0.25 mm × 30 m at a 28–500 amu scanning range; shunt rate at 50 mL/min; split ratio at 50:1; temperature setting in two stages (increase rate of 5 °C/min from 40–120 °C and increase rate of 10 °C/min from 120–200 °C) [29].

## 3. Results and Discussion

### 3.1. FT–IR Analysis

**Figure 1:**
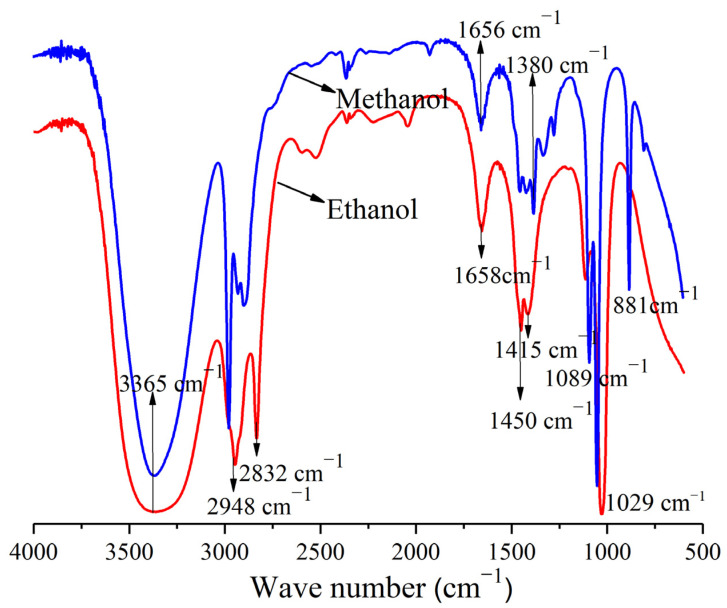
Fourier transform-infrared spectroscopic spectra of the ethanol and methanol samples of A. Bunge leaves.

The FT–IR spectrum of the ethanol and methanol extracted samples is shown in Figure 1. The absorption peaks of the two extracted samples were mainly concentrated at 3365–2948 cm^−1^, 2948–2832 cm^−1^, and 1658–881 cm^−1^. It shows that the organic chemical components contained in the sample include alcohols, ketones, and phenols [30,31]. As the characteristic absorption peak decreased, it indicated that compounds such as alcohols, phenols, and ketones were partially extracted [32]. According to the figure, the main structures of these lignins were very similar to those of the functional groups. All spectra showed a typical lignin pattern. The lignin characteristic absorption peaks occurred at 1658, 1450, 1415, and 1380 cm^−1^. No significant changes were observed at 1326, 1272, 1089, 1049, 1029, or 881 cm^−1^ [33]. However, the absorption intensity of the ethanol sample at 1658 cm^−1^ increased, but it decreased at other peaks, suggesting partial hydrolysis of cellulose lignin after extraction [34]. As the carbon species changed, the transmission intensity of all peaks gradually decreased, indicating that these groups contain less carbon [35].

The results showed that the main chemical functional groups were alcohol, fatty acid, and ether, which were also the main components of bio-oil. They can also synthesize a variety of biological medicine, pesticides, and cosmetics, and have a wide range of industrial applications. The change of functional groups reflects the oxidation activity of A. Bunge leaves to some extent. The aliphatic hydrogen and carbon groups in A. Bunge leaves are easier to decompose and precipitate in both pyrolysis and combustion. All C–H and C–O functional groups will precipitate together with the volatiles in the process of pyrolysis and combustion. In particular, the structure containing O and fat almost disappeared, indicating that intensity of absorption band in the corresponding band was weakened by varying degrees. The characteristic absorption peaks gradually decreased, indicating that some chemical components were extracted, including oxygen-containing, hydroxyl (–OH), aromatic ring compounds, etc. The results show that A. Bunge leaves have the potential of resource utilization and can be used as raw materials for bio-oil production.

### 3.2. GC–MS Analysis

**Figure 2:**
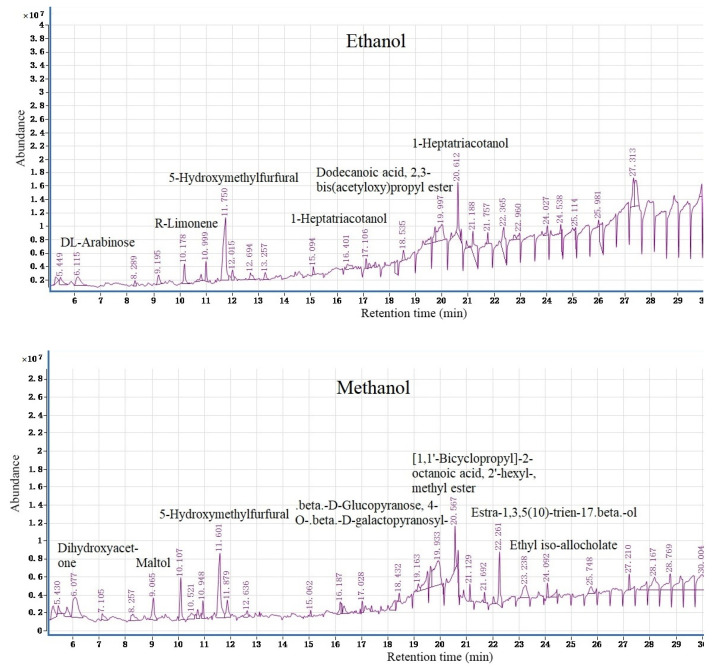
Total ion chromatogram of the A. Bunge leaves ethanol and methanol samples.

GC-MS results showed that 35 and 26 chemical components were identified in A. Bunge leaves ethanol and methanol extract samples (Figure 2), and the extraction rates were 55.35% (ethanol) and 39.82% (methanol), respectively (see the Tables S1 and S2 in the supporting material). Both extract samples contained seven identical chemical compositions (ethanol/methanol) including: DL-arabinose (1.73%/4.15%), 4H-pyran-4-one,2,3-dihydro-3,5-dihydroxy-6-methyl- (2.83%/1.77%), d-mannose (1.82%/0.74%), R-limonene (0.89%/1.15%), 5-hydroxymethylfurfural (7.33%/9.51%), melezitose (2.85%/1.84%), dodecanoic acid,3-hydroxy- (0.85%/0.41%) (see the Tables S1 and S2). In addition, the active ingredients detected include acetamide, 5-hydroxymethylfurfural, R-limonene, d-mannose, and dihydroxyacetone have good application prospects.

Acetamide is mainly used as an organic solvent, antacid in cosmetics production, and also used in preparation of sleeping pills and insecticides. Acetamide can synthesize new quinazoline derivatives, and new compounds can be used to prepare a standard drug for treating peptic ulcer and ulcerative colitis. Long-term oral administration of the compound had no side effects on liver and renal function [36]. 5-hydroxymethylfurfural (5-HMF) is an important furan compound, which is widely used in medicine, chemistry, energy, and other fields. 5-HMF can be used to detect the metabolites of glucose infusion [37]. 5-HMF has a protective effect on pharmacological applications to prevent angiocardiopathy and diabetic diseases [38]. R-limonene has insecticidal effect, and the percentage of macrophages infected by trypanosoma clarkii and the number of intracellular parasites decreased moderately at a concentration non-toxic to macrophages [39]. Limonene is a chemopreventive and clinically active food ingredient found in citrus peel oil. Limonene is widely distributed in human breast tissue and reduces the expression of cyclin D1 in breast cancer, resulting in reduced cancer cell proliferation [40]. D-mannose is a monosaccharide, which can be used in biochemical reagents and molecular biology. ArtinM is a d-mannose binding lectin, which can improve the immunogenicity of the lysate antigen of neosporidium. It has strong adjuvant and higher protective immune stimulation effect in neosporidium canis immunity [41]. Dihydroxyacetone is most commonly used in cosmetics, which can prevent excessive evaporation of water from the skin, moisturize the skin, and prevent ultraviolet radiation [42].

Through detailed analysis of the detected compounds, it was shown that the A. Bunge leaves of the plant contain many healthy and beneficial chemically active ingredients. The active compounds in the extract can be widely used in biomedical, chemical, and cosmetic fields. In addition, the extraction rate of ethanol is higher than that of methanol, and ethanol has fewer toxic side effects than methanol. Therefore, ethanol extraction can be preferred for future use in the pharmaceutical or cosmetic industry. GC–MS results can provide a correct scientific basis for the development and utilization of A. Bunge leaves.

### 3.3. PY/GC–MS Analysis

**Figure 3:**
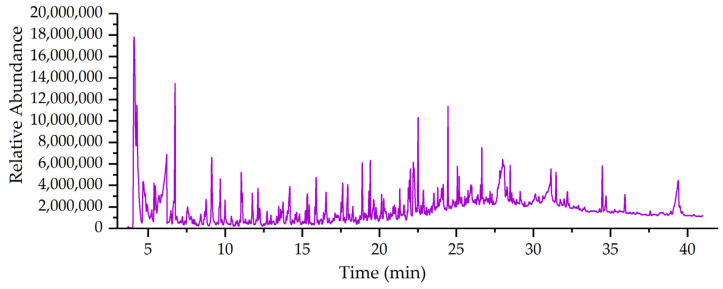
Total ion chromatograms of A. Bunge leaves by pyrolysis gas chromatography-mass spectroscopy.

All total of 269 chemical compositions were detected under PY/GC–MS detection analysis (see Table S3). The active components include: batilol, pregnenolone, benzoic acid, butyrolactone, and propanoic acid (Figure 3). Batilol promotes leukocyte proliferation to treat leucopenia caused by various diseases, such as leukopenia caused by radioactivity and antitumor drugs. The product is one of the commonly used drugs to promote leukocyte proliferation. The human hematopoietic system contains batilol, which may be a hematopoietic factor in the body. Batilol increases the number of granulocytes, and also has an anti-radiation effect, prevents leukopenia, and is used clinically as an antitumor drug for radiation therapy [43,44]. Pregnenolone protects the brain from overactivation of CB1 receptors and it is also an inactive precursor of all steroid hormones, which opens up methods to treat cannabis poisoning and addiction [45]. Pregnenolone reduces symptoms in people with severe schizophrenia, particularly in those who have not received a combination of mood stabilizers [46,47]. Benzoic acid has an inhibitory effect on molds, yeasts, and bacteria under acidic conditions, acts as a preservative and antimicrobial agent, and is mainly used as an antifungal and antiseptic [48,49]. Various compositions of analogues of 5,6,7-trihydroxyheptanoic acid have been used to treat posterior eye diseases and diseases characterized by excessive cell proliferation or angiogenesis [50].

Butyrolactone is an intermediate of the plant growth regulator butyric acid. It is used in the manufacturing of cyclopropylamine, pyrrolidone, and other drugs. Butyrolactone is an intermediate of synthetic insecticides, herbicides, vitamin B1, and chlorophyll and can also be used to synthesize plant growth regulators [51,52,53]. Propanoic acid is used as a preservative, anti-mold agent, spice, an intermediate of the bactericide metalaxyl, and also a pharmaceutical intermediate. The propanoic acid derivative-fluticasone propionate, combined with salmeterol-flutiacasone prevents chronic obstructive pulmonary disease and the pulmonary function, and quality of life of the patients were improved [54]. Tiotropium bromide combined with salmeterol/fluticasone propionate is clinically effective as an inhaled agent for patients with chronic obstructive pulmonary disease and has a good prognosis. It is worthy of clinical application [55].

The results of PY/GC−MS showed that the pyrolysis products could be used in biomedicine for 15.78%, including batilol, pregnenolone, benzoic acid, etc. The remaining 84.22% is basically used in the chemical industry, and there are many ingredients that can be used as chemical and industrial raw materials at the same time, such as acetic acid, formic acid, acetone, etc., (see Table S3). Bio-oil is a complex mixture of organic components with high oxygen content, including almost all kinds of oxygen-containing organic compounds, such as ketones, organic acids, alcohols, etc., [56]. According to the statistics in Table S3, these are classified into four classes: (1) aldehydes and ketones; (2) acids and esters; (3) alcohols and phenols; (4) hydrocarbons, with a high proportion of organic acids, aldehydes, and ketones. The results of this study showed that A. Bunge leaves contained a large amount of oxygenated organic mixtures in the pyrolysis products, which indicated that A. Bunge leaves could be used as raw materials for bio-oil extraction and had the potential to extract bio-oil.

## 4. Conclusions

In this study, the A. Bunge leaves was used as the research object to explore its potential for extracting bio-oil. The chemical composition of A. Bunge leaves identified by GC–MS was confirmed to contain a variety of healthy and beneficial chemicals, which could be used in biomedicine, and industrial and chemical applications. Further analysis by PY/GC–MS showed that rapid pyrolysis products contained a large number of oxygen-containing components, including ketones, alcohols, and organic acids, which were important components of bio-oil. The A. Bunge leaves could be used to extract bio-oil and had the potential to be new biomass energy source, which was conducive to the development and utilization of A. Bunge leaves. This is the first time that the pyrolysis technology is combined with A. Bunge leaves, and the extracts and pyrolysis products show the great value added products of A. Bunge leaves. Only a small number of experiments have been conducted in the laboratory so far, and the possibility of high yield of the bio-oil needs to be confirmed. Combining waste leaves with pyrolysis is an innovative method. In the future, various parameters of the pyrolysis method can be optimized to improve the pyrolysis efficiency and maximize the effect of pyrolysis to extract bio-oil.

## Data Availability

The data presented in this study are available on request from the corresponding author.

## Supplementary Materials

The following are available online at: https://www.mdpi.com/article/10.3390/polym14225003/s1. Table S1: Gas chromatography-mass spectroscopy analysis of the ethanol sample. Table S2: Gas chromatography-mass spectroscopy analysis of the methanol sample. Table S3: Pyrolysis/gas chromatography-mass spectroscopic analysis of A. Bunge leaves.
