# Supplementary material for: Pyrolysis of Aesculus chinensis Bunge Leaves as for Extracted Bio-Oil Material
Source: Polymers (Basel). 2022 Nov 18;14(22):5003. doi: 10.3390/polym14225003 (PMC9695936; doi:10.3390/polym14225003)
Supplement: Supplementary file 1 [file polymers-14-05003-s001.zip › polymers-1991002-supplementary.pdf]

Supplementary Materials for

Pyrolysis of *Aesculus chinensis* Bunge Leaves as for Extracted Bio-Oil Material

Yiyang Li [1,†], Qian Ma [1,†], Guanyan Li [1,†], Junwei Lou [2,*], Xiangmeng Chen [1], Yifeng He [1] and WanXi Peng [1,*]

1 Henan Province Engineering Research Center for Forest Biomass Value-Added Products, Henan Agricultural University, Zhengzhou 450002, China

2 School of Architectural Engineering, Zhejiang Business Technology Institute, Ningbo 315012, China

* Correspondence: 10815018@zjbti.net.cn (J.L.); pengwanxi@henau.edu.cn (W.P.)

† These authors contributed equally to this work

This file includes:

Table S1–3

Tables

Table S1. Gas Chromatography-Mass spectroscopy analysis of the ethanol sample.

No.	Retention Time (min)	Peak Area (%)	Component
1	5.24	1.73	DL-Arabinose
2	5.43	0.61	2-Hexanone, 6-hydroxy-
3	5.76	1.25	o-Acetyl-L-serine
4	6.08	4.95	Dihydroxyacetone
5	7.11	0.78	Melibiose
6	8.26	1.20	.beta.-D-Glucopyranose, 4-O-.beta.-D-galactopyranosyl-
7	9.07	2.15	Maltol
8	10.11	2.83	4H-Pyran-4-one, 2,3-dihydro-3,5-dihydroxy-6-methyl-
9	10.52	1.12	d-Mannose
10	10.75	0.70	d-Mannose
11	10.95	0.89	R-Limonene
12	11.60	7.33	5-Hydroxymethylfurfural
13	11.88	1.78	Melezitose
14	12.64	0.65	Melezitose
15	15.06	0.30	7-Methyl-Z-tetradecen-1-ol acetate

16	16.19	0.88	7-Methyl-Z-tetradecen-1-ol acetate
17	16.24	0.42	Melezitose
18	16.34	0.85	Dodecanoic acid, 3-hydroxy-
19	17.03	0.55	Acetamide, N-methyl-N-[4-(3-hydroxypyrrolidinyl)-2-butynyl]-
20	17.38	0.29	7-Methyl-Z-tetradecen-1-ol acetate
21	18.28	1.35	Acetamide
22	18.43	0.36	Acetamide, N-methyl-N-[4-(3-hydroxypyrrolidinyl)-2-butynyl]-
23	19.16	0.59	Desulphosinigrin
24	19.52	2.32	.beta.-D-Glucopyranose, 4-O-.beta.-D-galactopyranosyl-
25	19.64	1.53	.beta.-D-Glucopyranose, 4-O-.beta.-D-galactopyranosyl-
26	19.93	6.90	.beta.-D-Glucopyranose, 4-O-.beta.-D-galactopyranosyl-
27	20.12	0.23	.beta.-D-Glucopyranose, 4-O-.beta.-D-galactopyranosyl-
28	20.57	1.81	[1,1'-Bicyclopropyl]-2-octanoic acid, 2'-hexyl-, methyl ester
29	20.68	1.12	.beta.-D-Glucopyranose, 4-O-.beta.-D-galactopyranosyl-
30	21.13	0.60	Oxiraneundecanoic acid, 3-pentyl-, methyl ester, trans-
31	21.69	0.42	Ethyl iso-allocholate
32	22.03	0.42	Ethyl iso-allocholate
33	22.26	3.49	Estra-1,3,5(10)-trien-17.beta.-ol
34	23.24	2.30	Dasycarpidan-1-methanol, acetate (ester)
35	24.09	0.65	Ethyl iso-allocholate

Table S2. Gas Chromatography-Mass spectroscopy analysis of the methanol sample.

No.	Retention Time (min)	Peak Area (%)	Component
1	5.29	0.81	DL-Arabinose
2	5.45	1.20	DL-Arabinose
3	6.12	2.14	DL-Arabinose
4	8.29	0.41	Dodecanoic acid, 3-hydroxy-
5	9.20	1.11	Methyl 6-oxoheptanoate
6	10.18	1.77	4H-Pyran-4-one, 2,3-dihydro-3,5-dihydroxy-6-methyl-
7	10.81	0.74	d-Mannose
8	11.00	1.15	R-Limonene
9	11.75	9.51	5-Hydroxymethylfurfural
10	12.02	0.88	Melezitose
11	12.69	0.96	Melezitose
12	13.26	0.67	2-Myristynoyl pantetheine
13	15.09	0.47	1-Heptatriacotanol
14	16.40	1.18	1-Heptatriacotanol
15	17.11	0.51	1-Heptatriacotanol

16	17.22	0.40	1-Heptatriacotanol
17	17.46	0.36	1H-2,8a-Methanocyclopenta[a]cyclopropa[e]cyclodecen-11-one, 1a,2,5,5a,6,9,10,10a-octahydro-5,5a,6-trihydroxy-1,4-bis(hydroxymethyl)-1,7,9-trimethyl-, [1S-(1.alpha.,1a.alpha.,2.alpha.,5.beta.,5a.beta.,6.beta.,8a.alpha.,9.alpha.,10a.alpha.)]-
18	18.30	1.46	1H-2,8a-Methanocyclopenta[a]cyclopropa[e]cyclodecen-11-one, 1a,2,5,5a,6,9,10,10a-octahydro-5,5a,6-trihydroxy-1,4-bis(hydroxymethyl)-1,7,9-trimethyl-, [1S-(1.alpha.,1a.alpha.,2.alpha.,5.beta.,5a.beta.,6.beta.,8a.alpha.,9.alpha.,10a.alpha.)]-
19	18.54	0.79	1-Heptatriacotanol
20	18.96	2.22	1H-2,8a-Methanocyclopenta[a]cyclopropa[e]cyclodecen-11-one, 1a,2,5,5a,6,9,10,10a-octahydro-5,5a,6-trihydroxy-1,4-bis(hydroxymethyl)-1,7,9-trimethyl-, [1S-(1.alpha.,1a.alpha.,2.alpha.,5.beta.,5a.beta.,6.beta.,8a.alpha.,9.alpha.,10a.alpha.)]-
21	19.58	1.13	1-Heptatriacotanol
22	19.74	1.49	Dodecanoic acid, 2,3-bis(acetyloxy)propyl ester
23	20.00	4.53	Dodecanoic acid, 2,3-bis(acetyloxy)propyl ester
24	20.36	0.32	1-Heptatriacotanol
25	20.61	3.16	1-Heptatriacotanol
26	20.74	0.45	1-Heptatriacotanol

Table S3. Pyrolysis/Gas chromatography-mass spectroscopic analysis of A. Bunge leaves.

No.	Retention Time (min)	Peak Area (%)	Component
1	3.70	0.02	Octodrine
2	4.09	6.08	Ethyne, fluoro-
3	4.21	0.38	Carbon dioxide
4	4.27	3.96	Glycidol
5	4.67	0.82	Acetone
6	4.72	0.55	Propanal, 2-methyl-
7	4.80	0.61	Methyl glyoxal
8	4.90	0.12	Oxirane, trimethyl-
9	4.94	0.48	Formic acid
10	5.13	0.06	2-Propenoic acid
11	5.20	0.09	Propanal, 2-methyl-
12	5.24	0.17	2-Butenal, (E)-

13	5.38	0.33	Acetaldehyde, hydroxy-
14	5.46	0.51	2,3-Butanedione
15	5.70	0.31	Furan, 2-methyl-
16	5.85	0.02	Acetic acid
17	6.48	0.25	Butanal, 3-methyl-
18	6.65	0.44	2-Propanone, 1-hydroxy-
19	6.74	1.79	2-Propanone, 1-hydroxy-
20	6.85	0.17	Ethanone, 1-cyclopropyl-
21	7.10	0.08	2-Hexene, (Z)-
22	7.22	0.13	2-Pentanone, 3-methyl-
23	7.37	0.04	Oxalic acid, isobutyl octyl ester
24	7.42	0.04	Acetic acid, hydroxy-, methyl ester
25	7.56	0.45	Furan, 2,5-dimethyl-
26	7.63	0.20	Ethanol, 2-(vinyloxy)-
27	7.78	0.26	Propanoic acid
28	7.94	0.08	2-Propenoic acid
29	8.02	0.06	Butyric acid hydrazide
30	8.10	0.04	Thioacetic acid
31	8.41	0.30	1H-Pyrrole, 1-methyl-
32	8.54	0.02	Methylenecyclopropane
33	8.63	0.07	(E)-2-Butenylcyclopropane
34	8.66	0.19	Pyridine
35	8.76	0.51	Pyrrole
36	9.12	1.52	Toluene
37	9.47	0.10	Methyl Isobutyl Ketone
38	9.61	0.23	Propanoic acid, 2-oxo-, methyl ester
39	9.68	0.60	Propanoic acid, 2-oxo-, methyl ester
40	9.78	0.12	Cyclopentanone
41	9.99	0.45	2-Methyl[1,3,4]oxadiazole
42	10.09	0.02	2-Propenoic acid, 2-methyl-
43	10.11	0.03	Butyl aldoxime, 2-methyl-, syn-
44	10.40	0.16	3-Furaldehyde
45	10.46	0.07	5,5-Dimethyl-1,3-hexadiene
46	10.60	0.01	1,3-Cyclopentanedione
47	10.69	0.05	Pyridine, 2-methyl-
48	10.78	0.11	Pyrimidine, 2-methyl-
49	10.96	0.11	Propanenitrile
50	11.04	0.77	Furfural
51	11.11	0.41	2-Cyclopenten-1-one
52	11.30	0.15	Pyridine, 2,3,4,5-tetrahydro-
53	11.40	0.09	2-Butenoic acid, methyl ester, (Z)-
54	11.57	0.14	1H-Pyrrole, 2-methyl-

55	11.64	0.04	2-Hexenal
56	11.76	0.58	2-Furanmethanol
57	12.01	0.11	Ethylbenzene
58	12.13	0.52	2-Propanone, 1-(acetyloxy)-
59	12.24	0.39	Benzene, 1,3-dimethyl-
60	12.51	0.03	2-Heptyne
61	12.62	0.05	Phenol
62	12.72	0.22	Cyclopent-4-ene-1,3-dione
63	12.80	0.02	2,6-Lutidine
64	12.87	0.07	Carbonic acid, decyl prop-1-en-2-yl ester
65	12.96	0.15	Styrene
66	13.06	0.07	o-Xylene
67	13.14	0.08	Octanoic acid
68	13.35	0.16	2-Propenoic acid, 2-methyl-, oxiranylmethyl ester
69	13.47	0.29	2-Cyclopenten-1-one, 2-methyl-
70	13.60	0.28	Ethanone, 1-(2-furanyl)-
71	13.69	0.21	Butyrolactone
72	13.75	0.36	2(5H)-Furanone
73	13.96	0.18	1-Butene, 3-methyl-
74	14.05	0.25	Urea, 1-methylcyclopropyl-
75	14.19	0.90	2-Cyclopenten-1-one, 2-hydroxy-
76	14.35	0.09	Phenol, 3,5-dimethyl-
77	14.42	0.06	Pyridine, 3,5-dimethyl-
78	14.55	0.20	2(5H)-Furanone, 5-methyl-
79	14.63	0.18	2,5-Furandione, dihydro-3-methylene-
80	14.82	0.30	2-Pentanone, 3-methylene-
81	15.04	0.08	Benzene, [(methoxymethoxy)methyl]-
82	15.13	0.04	7-Oxabicyclo[4.1.0]heptane, 1-methyl-
83	15.20	0.17	2-Butanone, 3,3-dimethyl-
84	15.33	0.55	2-Furancarboxaldehyde, 5-methyl-
85	15.45	0.24	2-Cyclopenten-1-one, 3-methyl-
86	15.55	0.04	Pyridine, 4-ethenyl-
87	15.66	0.02	2H-Pyran-2-one
88	15.71	0.02	Furyl hydroxymethyl ketone
89	15.82	0.18	4(1H)-Pyrimidinone, 6-methyl-
90	15.90	0.80	Phenol
91	16.02	0.07	4-Cyclopentene-1,3-dione
92	16.15	0.05	1-Decene
93	16.22	0.12	Heptanoic acid
94	16.38	0.16	Benzene, 1,2,4-trimethyl-
95	16.46	0.13	2H-Pyran-2,6(3H)-dione
96	16.55	0.67	2-Methyliminoperhydro-1,3-oxazine

97	16.74	0.14	1-Heptene, 6-methyl-
98	16.93	0.08	dl-Threitol
99	17.02	0.12	1H-Pyrrole-2-carboxaldehyde
100	17.13	0.28	4(1H)-Pyrimidinone, 6-hydroxy-
101	17.24	0.16	Glycerin
102	17.31	0.10	dl-Threitol
103	17.37	0.11	Benzene, 1,2-diethyl-
104	17.44	0.07	Benzene, 2-propenyl-
105	17.52	0.26	D-Limonene
106	17.63	0.70	1,2-Cyclopentanedione, 3-methyl-
107	17.74	0.15	Benzyl alcohol
108	17.83	0.05	2,5-Furandione, 3,4-dimethyl-
109	17.95	0.68	Methylamine, N,N-dimethyl-
110	18.06	0.27	4-Methyl-5H-furan-2-one
111	18.28	0.37	Phenol, 2-methyl-
112	18.40	0.08	2(3H)-Furanone, dihydro-4-methyl-
113	18.47	0.08	2-Cyclopenten-1-one, 2-hydroxy-3,4-dimethyl-
114	18.58	0.03	2-Butanone, 4-cyclopentylidene-
115	18.63	0.04	Ethanone, 1-(1H-pyrrol-2-yl)-
116	18.75	0.15	Acetophenone
117	18.81	0.04	Benzaldehyde, 2-methyl-
118	18.89	0.89	p-Cresol
119	19.04	0.18	2-Cyclopenten-1-one, 3-ethyl-
120	19.13	0.13	Heptanoic acid
121	19.16	0.08	3-Ethyl-1,5-octadiene
122	19.25	0.10	Methyl 2-furoate
123	19.33	0.39	Hydrouracil, 1-methyl-
124	19.41	0.71	Phenol, 2-methoxy-
125	19.64	0.76	sec-Butylamine
126	19.78	0.26	11-Dodecen-1-yl acetate
127	19.86	0.14	Acetamide, N-(4-amino-3-furazanyl)-2-(3-quinuclidinyloxy)-
128	19.98	0.04	Benzofuran, 2-methyl-
129	20.04	0.09	(E)-3(10)-Caren-4-ol
130	20.15	0.47	Maltol
131	20.29	0.34	2-Cyclopenten-1-one, 3-ethyl-2- Furan hydroxy-
132	20.35	0.24	4-Methyl-1-prop-1-ynyl-cyclohexanol
133	20.43	0.22	4-Pyridinol
134	20.62	0.21	Phenol, 3,5-dimethyl-
135	20.74	0.13	Benzyl nitrile
136	20.80	0.09	2(3H)-Benzofuranone, hexahydro-3-methylene-
137	20.84	0.07	9-Octadecen-1-ol, (Z)-

138	20.89	0.17	Phenol, 3,5-dimethyl-
139	20.97	0.27	4H-Pyran-4-one, 2,3-dihydro-3,5-dihydroxy-6-methyl-
140	21.04	0.29	2H-Pyran-2-one, tetrahydro-
141	21.24	0.20	2-(E)-Pentenoic acid, (4S)-amino-5-phenyl-, ethyl ester
142	21.33	0.34	Phenol, 4-ethyl-
143	21.38	0.12	Phenol, 2,4-dimethyl-
144	21.43	0.09	11-Tetradecen-1-ol, (E)-
145	21.51	0.10	Octanoic acid
146	21.61	0.38	Benzoic acid
147	21.82	0.14	1-Decanol, 2-hexyl-
148	21.88	0.39	Ethanone, 1-(2-methylphenyl)-
149	21.95	0.57	4-Hydroxy-N-methylpiperidine
150	22.02	0.61	Creosol
151	22.11	0.07	Methyl salicylate
152	22.21	1.98	Catechol
153	22.40	0.20	Catechol
154	22.52	1.58	Benzofuran, 2,3-dihydro-
155	22.70	0.31	Dichloroacetic acid, undec-2-enyl ester
156	22.78	0.05	Pentadecanenitrile
157	22.86	0.68	1,2-Dimethyl cyclopropene
158	23.00	0.16	2,6-Dimethyl-4-thiopyrone
159	23.06	0.17	Benzenepropanenitrile
160	23.15	0.12	9-Borabicyclo[3.3.1]nonane, 9-ethyl-
161	23.19	0.22	2-Cyclohexen-1-one, 3,4,4-trimethyl-
162	23.31	0.20	3,9-Epoxytricyclo[4.2.1.1(2,4)]decan-10-one, 9-methyl-
163	23.39	0.21	Naphthalene, 1,2-dihydro-6-methyl-
164	23.55	0.76	1,2-Benzenediol, 3-methoxy-
165	23.62	0.08	1H-Indene, 1,1-dimethyl-
166	23.66	0.16	Hex-4-enoic acid, ethyl ester
167	23.79	0.58	Phenol, 4-ethyl-2-methoxy-
168	23.85	0.20	Decane, 1-fluoro-
169	23.92	0.27	Hydroquinone
170	23.96	0.19	Hydroquinone
171	24.05	0.90	1,2-Benzenediol, 4-methyl-
172	24.15	0.68	Indole
173	24.31	0.25	Cyclodecylamine
174	24.46	1.38	2-Methoxy-4-vinylphenol
175	24.59	0.27	Nerolidyl propionate
176	24.63	0.12	1,2-Dipropylcyclopropene-3-carboxylic acid

177	24.77	0.60	1,3-Benzenediol, 2-methyl-
178	24.83	0.23	p-Toluenesulfinic acid
179	24.88	0.17	3-Buten-2-one, 3-methyl-4-(1,3,3-trimethyl-7-oxabicyclo[4.1.0]heptan-1-yl)-
180	24.96	0.33	(Z)-4-Decen-1-ol, trifluoroacetate
181	25.07	0.74	Phenol, 2,6-dimethoxy-
182	25.18	0.60	Phenol, 2-methoxy-3-(2-propenyl)-
183	25.24	0.24	2-Fluorophenethyl alcohol, isopropyl ether
184	25.31	0.61	2-Heptanone, 3-propylidene-
185	25.55	0.45	Cyclopentadecanone, 2-hydroxy-
186	25.67	0.48	Octadecane, 1-bromo-
187	25.75	0.35	Indole, 3-methyl-
188	25.94	1.18	Vanillin
189	26.00	0.39	Phenol, 2-methoxy-5-(1-propenyl)-, (E)-
190	26.04	0.27	Methyl 6-O-[1-methylpropyl]-.beta.-d-galactopyranoside
191	26.11	0.21	9-Methyltricyclo[4.2.1.1(2,5)]deca-3,7-diene-9,10-diol
192	26.21	0.19	Bicyclo[3.1.0]hexane-6-methanol, 2-hydroxy-1,4,4-trimethyl-
193	26.25	0.29	3-buten-2-one, 4-(5,5-dimethyl-1-oxaspiro[2.5]oct-4-yl)
194	26.35	0.35	Phenol, 4-(methoxymethyl)-
195	26.40	0.56	Ethyl mandelate
196	26.57	0.57	3,5-Dimethoxy-4-hydroxytoluene
197	26.65	0.85	trans-Isoeugenol
198	26.73	0.43	Ethyl mandelate
199	26.84	0.30	3-Pyridinecarbonitrile, 2-methoxy-4,6-dimethyl-
200	26.89	0.17	Cyclododecane, ethyl-
201	26.93	0.38	O-(4-Fluorophenyl) diethylthiocarbamate
202	26.99	0.22	Pentacyclo[9.1.0.0(2,4).0(5,7).0(8,10)]dodecane, 3,3,6,6,9,9,12,12-octamethyl-, anti,syn,anti-
203	27.08	0.52	2- Chloropropionic acid, hexadecyl ester
204	27.19	0.37	Pentadecane
205	27.25	0.19	Benzonitrile, 2,4,6-trimethyl-
206	27.31	0.34	5-Hepten-3-yn-2-ol, 6-methyl-5-(1-methylethyl)-
207	27.41	0.39	Pyridine, 5-ethenyl-2-methyl-
208	27.68	1.04	8-Bromooctanoic acid
209	27.82	1.42	3-Methoxythiobenzamide
210	28.01	3.78	.beta.-D-Glucopyranose, 1,6-anhydro-
211	28.23	0.40	Dodecanoic acid
212	28.30	0.70	Nonanoic acid, 1-methylethyl ester
213	28.50	0.79	2,3,5,6-Tetrafluoroanisole

214	28.59	0.41	Cyclopentanemethanol, 1-hydroxy-.alpha.,3,3-trimethyl-2-(3-methyl-1,3-butadienyl)-
215	28.70	0.41	Pregnenolone
216	28.76	0.18	3-buten-2-one, 4-(5,5-dimethyl-1-oxaspiro[2.5]oct-4-yl)
217	28.82	0.22	Estra-1,3,5(10)-trien-17.beta.-ol
218	28.91	0.33	1-Cyclohexene-1-methanol, .alpha.,2,6,6-tetramethyl-
219	28.98	0.26	2,5-Dimethoxyethylbenzene
220	29.04	0.23	Panaxjapyne A
221	29.14	0.84	trans-Ferulic acid
222	29.43	0.32	2-[Ethyl(methyl)amino]ethyl cyclohexyl(hydroxy)phenylacetate
223	29.73	0.72	Batilol
224	29.78	0.29	Cyclopentadecanone, 2-hydroxy-
225	29.88	0.15	2-Dodecen-1-yl(-)succinic anhydride
226	29.96	0.35	9-Undecen-2-one, 6,10-dimethyl-
227	30.09	0.48	1(2H)-Naphthalenone, 3,4,4a,7,8,8a-hexahydro-2-hydroxy-8,8-dimethyl-, (2.alpha.,4a.beta.,8a.beta.)-
228	30.13	0.68	4a(2H)-Naphthalenecarboxylic acid, octahydro-, cis-
229	30.27	0.20	Estra-1,3,5(10)-trien-17.beta.-ol
230	30.36	0.59	11-Isopropylidene-tricyclo[4.3.1.1(2,5)]undecan-10-ol
231	30.62	0.56	2-Butenoic acid, 4-hydroxy-, methyl ester
232	30.67	0.18	5-Nonanone, O-methyloxime
233	30.75	0.30	2-Hydroxy-1,1,10-trimethyl-6,9-epidioxydecalin
234	30.88	0.81	2-Trimethylsilyl-1,3-dithiane
235	31.13	2.48	(E)-2,6-Dimethoxy-4-(prop-1-en-1-yl)phenol
236	31.47	0.91	Cycloundecane, (1-methylethyl)-
237	31.75	0.31	Thiazolo[4,5-f]quinoline
238	31.80	0.19	1-Hexadecyne
239	32.00	0.54	cis-Z-.alpha.-Bisabolene epoxide
240	32.21	0.58	Tetradecanoic acid
241	32.31	0.51	2(1H)-Naphthalenone, octahydro-4a-methyl-7-(1-methylethyl)-, (4a.alpha.,7.beta.,8a.beta.)-
242	32.61	0.32	9-Undecen-2-one, 6,10-dimethyl-
243	32.78	0.22	.alpha.-Methyl mannofuranoside
244	32.93	0.24	2-Butenal, 3-methyl-, dibutylhydrazone
245	33.04	0.16	Z-(13,14-Epoxy)tetradec-11-en-1-ol acetate
246	33.21	0.10	2-Dodecen-1-yl(-)succinic anhydride
247	33.30	0.24	Cyanamide, dibutyl-
248	33.73	0.12	9-Undecen-2-one, 6,10-dimethyl-
249	34.30	0.13	2-Dodecen-1-yl(-)succinic anhydride
250	34.48	0.71	Neophytadiene

251	34.71	0.41	2-Pentadecanone, 6,10,14-trimethyl-
252	35.10	0.03	.beta.-D-Glucopyranoside, 1-O-methyl-4,6-O-n-hexylidene-
253	35.28	0.06	2-Dodecen-1-yl(-)succinic anhydride
254	35.38	0.03	Docosanoic acid
255	35.58	0.03	8-Hexadecenal, 14-methyl-, (Z)-
256	35.94	0.29	Tetradecanal
257	36.26	0.03	Erucic acid
258	36.49	0.04	Cyclopentanone, 3-(6,6-dimethyl-5-oxo-2-heptenyl)-, (E)-
259	36.73	0.06	2-Dodecen-1-yl(-)succinic anhydride
260	37.58	0.08	2-Dodecen-1-yl(-)succinic anhydride
261	37.75	0.01	4-(methylthio)benzylmethylketoxime
262	38.27	0.11	2-Dodecen-1-yl(-)succinic anhydride
263	38.35	0.09	2-Dodecen-1-yl(-)succinic anhydride
264	38.64	0.06	2-Dodecen-1-yl(-)succinic anhydride
265	38.93	0.12	1,13-Tetradecadiene
266	39.40	1.89	n-Hexadecanoic acid
267	39.50	0.23	2-Decene, 3-methyl-, (Z)-
268	39.66	0.11	Dibutyl phthalate
269	40.42	0.05	4a,7,7,10a-Tetramethyldodecahydrobenzo[f]chromen-3-ol
